# In Situ Mechanical Analysis of the Nanoscopic Solid Electrolyte Interphase on Anodes of Li‐Ion Batteries

**DOI:** 10.1002/advs.201900190

**Published:** 2019-06-14

**Authors:** Boaz Moeremans, Hsiu‐Wei Cheng, Claudia Merola, Qingyun Hu, Mehtap Oezaslan, Mohammadhosein Safari, Marlies K. Van Bael, An Hardy, Markus Valtiner, Frank Uwe Renner

**Affiliations:** ^1^ Institute for Materials Research Hasselt University BE‐3590 Diepenbeek Belgium; ^2^ Max‐Planck Institut für Eisenforschung GmbH 40237 Düsseldorf Germany; ^3^ Institut für Physikalische Chemie II TU Bergakademie Freiberg 09599 Freiberg Germany; ^4^ Institute for Applied Physics Applied Interface Physics Technical University of Vienna 1040 Vienna Austria; ^5^ Physical Chemistry Electrocatalysis Carl von Ossietzky University of Oldenburg 26111 Oldenburg Germany; ^6^ IMEC Division IMOMEC BE‐3590 Diepenbeek Belgium

**Keywords:** Li‐ion batteries, polymers, soft matter, solid–electrolyte interphases, surface force apparatus

## Abstract

The interfacial decomposition products forming the so‐called solid–electrolyte interphase (SEI) significantly determine the destiny of a Li‐ion battery. Ultimate knowledge of its detailed behavior and better control are required for higher rates, longer life‐time, and increased safety. Employing an electrochemical surface force apparatus, it is possible to control the growth and to investigate the mechanical properties of an SEI in a lithium‐ion battery environment. This new approach is here introduced on a gold model system and reveals a compressible film at all stages of SEI growth. The demonstrated methodology provides a unique tool for analyzing electrochemical battery interfaces, in particular in view of alternative electrolyte formulations and artificial interfaces.

Obtaining control of the solid electrolyte interphase (SEI) is key for ultimately advancing the life‐time of lithium‐ion batteries.^1^, ^2^ Yet, the internal structure and composition of this nanoscopic reduction‐product layer is complex. The considerably increasing requirements for cost, safety, rate capability, and cycle life, rejuvenate currently the ultimate interest in controlling the interfacial properties in lithium‐ion batteries. Li ions become immobilized during the SEI formation in the form of various lithium compounds. Also the rate capability depends critically on the forming interface layer as well as for example the integrity of the active anode particles. The introduction of electrolyte additives that influence the SEI formation on graphitic anodes^1^, ^2^, ^3^ have improved the battery performance significantly over the years. Recently, new (composite) Li‐alloy anodes offer a significant increase in stored energy density compared to state‐of‐the‐art graphitic materials but pose enormous demands on the interface stability due to much larger volume changes. Next to commercially promising Si or Sn alloys, also Ag or Au are eventually used as coatings and frequently addressed as model systems. The continuous degradation of the SEI on alloying materials has been recognized as their key problem, currently preventing their full commercial introduction.^4^, ^5^, ^6^ A customized electrochemical surface force apparatus (SFA) is used here to control the growth and to investigate the mechanical properties of the SEI in a lithium‐ion battery environment. We utilize in this study Au thin films on mica, yet any other metal thin film with a film roughness below the expected film growth, or graphene to mimic graphite basal surfaces,^7^ may be used. SFA force spectroscopy on the forming SEI on Au reveals a compressible film at all stages of growth. The structure is in line with a thin inorganic–organic inner rigid layer and an outer polymeric and eventually porous layer including precipitates. Employing white‐light interferometry in the SFA combined with force spectroscopy, we obtain insight in the composition and multilayer structure of the SEI and challenge classic views. The demonstrated methodology provides a unique tool for analyzing electrochemical battery interfaces, in particular in view of alternative electrolyte formulations and artificial interfaces.

To improve the solid electrolyte interphase on any active anode material, it is vital to understand its structure, composition, and formation mechanism. The various factors which influence the SEI are the chemical parameters such as the type of electrolyte, active material, binder, and the conductive material, but also temperature and battery cycling conditions have a profound influence. A thorough characterization remains challenging due to the nanoscopic structure and its inherent 3D inhomogeneity.^8^, ^9^, ^10^, ^11^, ^12^, ^13^, ^14^, ^15^, ^16^ The thickness of the forming SEI is a critical experimental and theoretical parameter^17^, ^18^, ^19^, ^20^, ^21^ and early models included polyhetero‐microphases of inorganic and organic compounds^17^ and multilayer models[qv: 9,16,]^18^

Experimentally, postmortem sample treatment may heavily influence measured thicknesses^22^ as well as risk beam damage during spectroscopic analysis.^23^ In situ atomic force microscopy (AFM)^24^, ^25^, ^26^, ^27^, ^28^, ^29^, ^30^ is able to directly address thickness and surface topography and related mechanical analysis but is hampered by the typical sharp tip geometries even if colloidal probes are used.^30^ In contrast, sampling over a diameter of tens of micrometers, an SFA^31^, ^32^ enables to analyze both, the average absolute growth and the mechanical compressibility of the SEI on an alloying anode material (**Figure** **1**c). During an SFA experiment, two opposing materials on crossed, cylindrical silica or mica disks form an atomically smooth contact and create an interferometer. The opposing surface is an atomically smooth gold mirror (or any other metal) in contact with electrolyte, prepared using template stripping.^7^, ^31^ The generated interference patterns—so‐called fringes of equal chromatic order (FECO)—reveal the distance (Δ*D*) between the two mirrors with a sub‐Ångstrom resolution as well as the applied forces. The electrolyte used for our experiments reported here was 1 m LiPF_6_ in ethylene carbonate (EC) equally mixed by volume with diethylene carbonate (DEC), with additional fluoro‐ethylene carbonate (10 wt%) and vinylene carbonate (2 wt%). After a dry contact is established and the SFA‐box was filled with battery electrolyte, two different types of galvanostatic SFA experiments were conducted. In a first approach, the SEI growth was monitored by manually compressing the interface during the electrochemical experiment. In the second approach, the galvanostatic current was applied until predefined voltages, i.e., different depths of charge (DoC), were reached, after which force experiments were conducted on the freshly electrochemically modified surfaces. Thus, the evolution of the mechanical properties of the SEI revealed by force measurements can be monitored together with the absolute thickness. The thickness is sensed by the point of initial compression and the final hard wall of compressed SEI.

**Figure 1:**
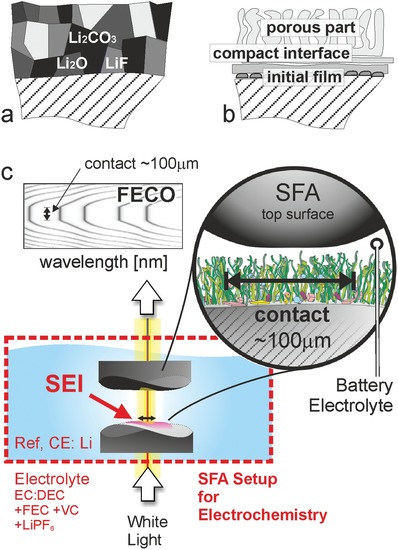
SEI model structures and battery‐SFA setup. SEI models suggested by a) Peled^17^ and b) Aurbach.^18^ c) Experimental setup of the electrochemical surface forces apparatus (SFA), modified for lithium‐ion battery testing (battery‐SFA).

**Figure** **2** shows the mirror shift together with the directly corresponding voltage profile, monitoring the initial SEI growth on a pristine Au sample surface as measured by the SFA. The distance between the two mirrors slowly relaxes to finally 4 nm after the contact is closed, in line with the earlier observed wetting phenomena.^7^ The galvanostatic experiment is started after opening the contact between the sample mirror surfaces, and a constant current of −20 mA is applied. The initial limits of the voltage were 0.4 and 3.0 V versus Li/Li+, completing the first discharge. After each cycle, a rest period is maintained for 100 s before the next cycle is commenced. After four cycles, the lower voltage limit was removed and the current reversed after 1000 s. Visibly, no electrolyte reduction product deposition occurs at 2.5 V versus Li/Li+, at the gold electrode during the initial 4000 s. With the potential then dropping well below 2.5 V during the first charge cycle, the mirror shift starts to increase to 12, 16, and then 20 nm, indicating the growth of an SEI.

**Figure 2:**
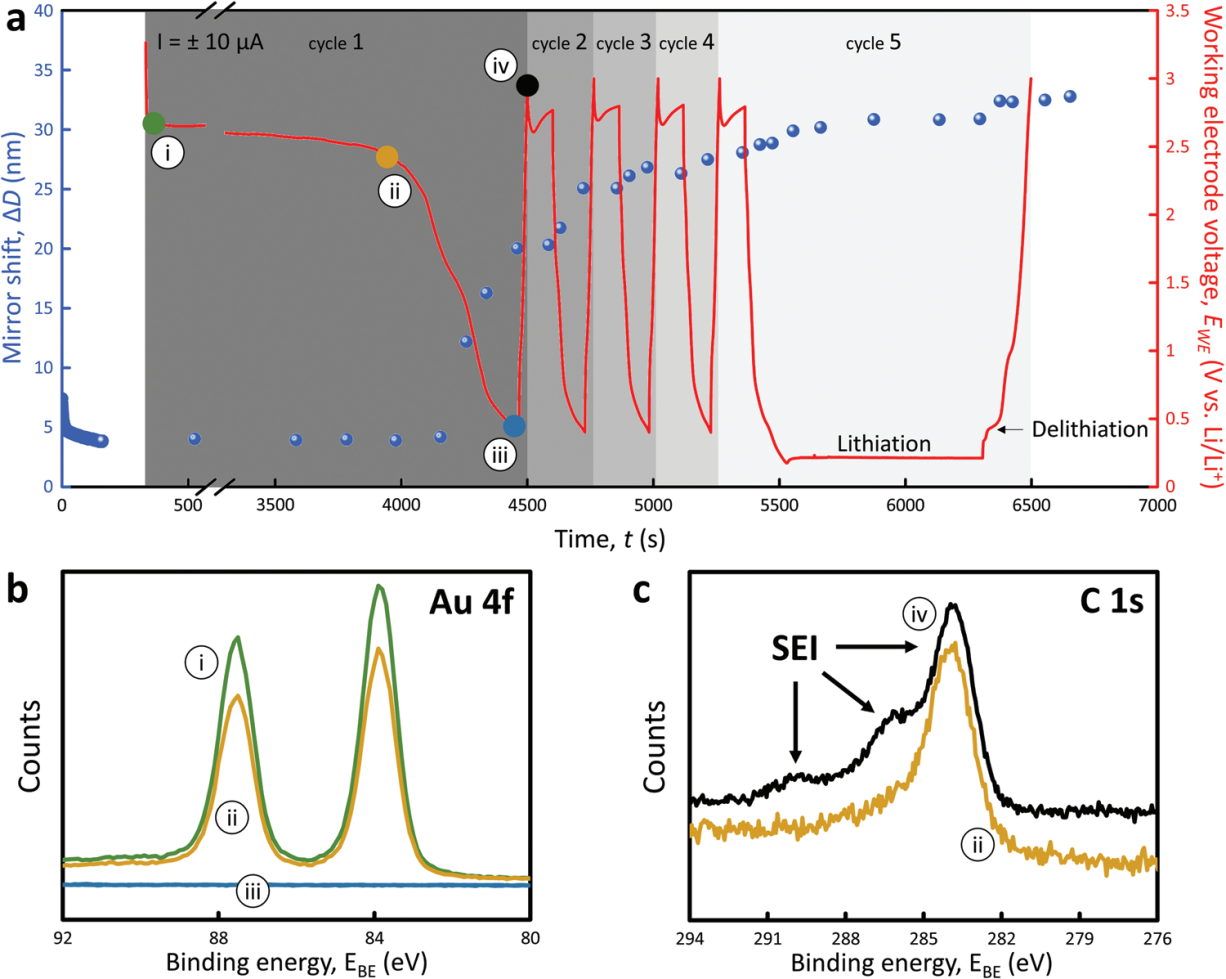
SEI formation followed by in situ SFA and XPS. a) Mirror shift evolution (SEI growth, blue dots) and working electrode evolution of voltage (red line) during galvanostatic discharge of the Au surface. The colored dots (i–iv: green, blue, orange, and black) correspond with the points at which the experiment was stopped and the b) high‐resolution Au 4f XPS scans were recorded. At similar stages c) extended XPS analysis on a flat gold coated mica surface was performed.

The existence of a new reaction layer was also confirmed using X‐ray photoelectron spectroscopy (XPS). High‐resolution Au 4f spectra from the Au substrate are displayed in Figure 2b. At 2.6 and 2.3 V versus Li/Li+, the Au 4f peaks are clearly visible i,ii). However, at 0.4 V iii) and after one complete cycle iv), the Au photoelectrons cannot be detected anymore indicating an electron‐blocking film of at least 10 nm. During the first four cycles, the SEI keeps growing with the SFA intermirror distances increasing to 27 nm. As the voltage limit is removed, here in the fifth cycle, the voltage drops to 0.2 V versus Li/Li+ and forms a plateau caused by the lithiation of gold forming Li–Au alloys.^33^ This lithiation does not occur directly at the contact spot, but in the less confined direct surrounding. After minor growth at the beginning of the alloying reaction, the SEI thickness remains rather stable during the rest of the gold lithiation. Upon reversing the current, during the dealloying reaction, the SEI grows an additional 1.5 nm. Finally, an intermirror distance of 33 nm is reached. A typical SEI signature is obtained in extended XPS measurements performed in control experiments on the same but flat substrates (Figure 2c‐ii,iv). The spectra obtained after electrochemistry in a glovebox and direct transfer into the XPS setup are identical to previously reported SEI on Au.

After the manual approach as performed above, the distance between sample and probe mirror relaxes. The exact approach curve and relaxing behavior depend on the approach speed (which is not constant in the manual approach) while the final hard wall (minimum thickness under maximum compression) is independent of the movement. **Figure** **3** shows force–distance characteristics during well‐controlled motorized approach at different DoC. In all five displayed force characteristics, the force necessary to move the surfaces toward each other increases until it hits a hard wall. At this point, the two disks cannot be moved further toward each other, even at higher applied loads. The position of this hard wall prior to the electrochemical experiment (at open circuit voltage—red dots) is 4 nm, the same value which was obtained during the “manual compression” experiment (Figure 2a) and similar to simple immersion^7^ indicating an electrolyte layering structure. At 2.3 V versus Li/Li+, the voltage drop due to the SEI formation has just initiated before the experiment was stopped. This results in a slight increase of the hard wall position at 4.5 nm.

**Figure 3:**
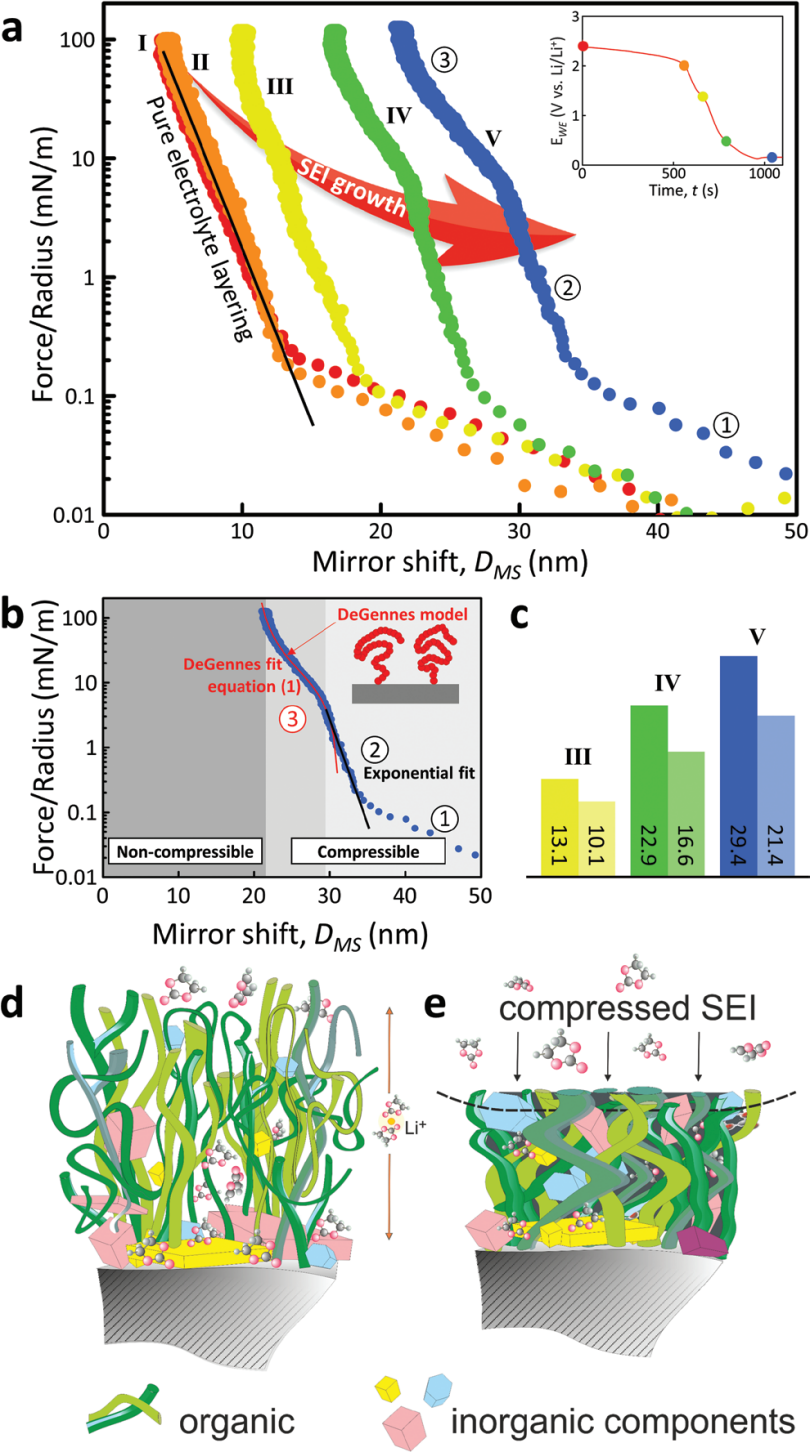
In situ SFA force spectroscopy of SEI and analysis. a) Force run curves on the SEI on gold‐coated mica, taken on different depths of discharge, indicated by voltage–time curve (inset). b) De Gennes fitting performed on a force run curve. The gray areas indicate the thickness of the SEI prior and after compressing. c) SEI thickness values prior and after compressing are plotted for three different DoC. Model structure sketches of the SEI d) prior and e) after compression.

A clear signature for a new compressible film can be recognized in the force–distance characteristic shown in Figure 3a. In the subsequent force runs, the hard wall position increases to 10 nm (at 1.3 V vs Li/Li+), 17 nm (0.5 V), and finally 22 nm for the starting lithiation, indicating the same growth pattern as observed in the manual compression shown in Figure 2a. The total compressibility of the latter SEI films is larger compared to initial curves and finally constant, indicating the relatively more important presence of a rigid film component in the thinner initial layer. Yet, there is a clear compressibility of the SEI film even in the very initial stage, pointing to a considerable contribution of polymeric components from the beginning of the SEI growth. The slopes do allow for the deeper analysis of the mechanical properties, i.e., the elastic modulus, but the purpose here is to follow the growth in situ.

The recorded force profiles display three major additive contributions in line with established interpretations in terms of the DLVO theory:^34^, ^35^ 1) A long range electric double layer interaction, 2) a short range interfacial electrolyte layer compression (in other systems also termed as solvation interaction), and 3) SEI compressibility, as shown in detail in Figure 3b. During the initial approach at all potentials, an exponential increase in force is observed until a force of about 0.2 mN m^−1^, where a sudden change of the exponential slope is observed. This turning point indicates the start of more structured electrolyte layering.^7^ This behavior is due to the compression and pushout of the molecules in the liquid contact zone, characterized by steric repulsion due to electrolyte confinement. Typically at forces >3–5 mN m^−1^, a characteristic change of the force profile indicates SEI compressibility, which does not display a constant exponential slope. Specifically, Figure 3b shows the fitting of the force run taken at 0.1 V versus Li/Li+. Here, the SEI compression part of the force–distance profile can be fitted well using an empirically modified De Gennes equation^34^, ^35^ for compressing grafted polymers

(1) $$F\left(D\right)/R=\frac{2\pi kT}{s^{3}}\left[\frac{4}{5}\left(D-D_{0}^{\mathrm{dG}}\right){\left(\frac{2L}{\left(D-D_{0}^{\mathrm{dG}}\right)}\right)}^{9/4}-\frac{4}{7}\left(D-D_{0}^{\mathrm{dG}}\right){\left(\frac{\left(D-D_{0}^{\mathrm{dG}}\right)}{2L}\right)}^{3/4}\right]$$

Here, F(*D*)/*R* is the force normalized by the radius, *D* the substrate movement (or mirror shift), $D_{0}^{\mathrm{dG}}$ the shift away from the plane of origin, *L* the compressibility of the polymer, and *s* the effective average grafting density, which indicates the average distance between two anchor points of the polymer chains on the substrate.

The applicability of De Gennes equation (Equation (1)) is indeed pointing to grafted polymer structures as a base model for SEI. A more complex analysis of the data curves obtained from organic–inorganic composite layers will require the further development of the theory and is not in the scope of this communication. Figure 3c shows the SEI thickness at low load and at highest applied loads. The values of the effective compressibility *L* for the force runs performed at 1.3, 0.5, and 0.2 V versus Li/Li+ remain relatively constant at about 23% of the layer thickness during the very beginning of growth and slightly higher 27% for the further stages (regions indicated in Figure 3b), as shown in Figure 3c. Thus, interestingly, no large difference in early and later growth was obtained by SFA, which is pointing to a simultaneous evolution from the start of both the layer interfacing the electrode and the layer close to the electrolyte. A kinetic analysis of the recorded current profiles (shown in Figures S1–S4 in the Supporting Information) is indicating both organic and inorganic components.

Although the SEI is a complex mixture of inorganic and organic compounds, it is noteworthy that the obtained effective grafting density in these force runs (2–3 nm) is very similar to grafting densities obtained in similar experiments with grafted polymer brushes. As such, a mostly inorganic mosaic structure of the SEI can be ruled out even at early stages of growth. On the contrary, a layered, eventually laterally inhomogeneous SEI model with a more compact inner interphase and a flexible outer part does explain the observed force profiles. A number of different as well as similar models have been suggested before.^9^, ^16^, ^17^, ^18^, ^28^ In summary, the combined data analysis of in operando SFA and kinetic data therefore suggests a layered model as illustrated in Figure 3d.

This result demonstrates that SEIs are to a large volume fraction polymeric and potentially flexible in nature. Tuning of polymeric structures will hence provide a viable strategy for optimizing performance during large volume expansion and contraction cycles. The proposed methodology will serve as a unique and valuable new tool for analyzing and ultimately tuning the mechanical properties of SEI layers by novel additives or electrolyte formulations. Both compressibility and average grafting densities along with long‐term measurements may serve as effective parameters for understanding and tuning the SEI performance over the life‐cycle of a battery, from initial formation to structural changes during extended cycling, and their mechanical properties can be assessed in real time.

## Experimental Section

*SFA Setup*: A surface force apparatus (SFA‐2000, SurForce LLC) setup was used in this study. Two semitransparent curved disks were placed in a crossed cylinder geometry in an argon atmosphere inside an airtight steel box. The top disk was mechanically fixed. The bottom disk was attached to a spring and displacement mechanics, which allowed to move the opposing disks into a well‐defined contact at a given force. The contact region can flatten and comply with the opposing surface due to the glue that was used to fix mica sheets on the silica disks. During the experiment, white light was guided through these disks, which both had a semitransparent mirror: silver on the backside of mica, i.e., not in contact with electrolyte, and the gold thin film working electrode disk. When moved close together, these mirrors formed an interferometer. The constructive and destructive interference of the white light at discrete wavelengths led to the generation of the fringes of equal chromatic order, which were detected by guiding the interfered light into a grating spectrometer using a set of mirrors. A typical FECO is shown in Figure 1c, which clearly depicts the flat region of fringes indicating an extended flat contact region. The FECO allowed the determination of the intermirror distance with a nominal resolution of 10–30 pm, i.e., well below 1 Å. At the same time, the lateral shape of the fringes represented an image of a segment of the contact area, having a lateral resolution of ≈1.0 µm. The flat round‐shaped contact area of the mica–Au setup had a diameter of about 50–100 µm. During the initial contact situation in dry argon atmosphere, the absolute zero distance (DMS = 0) was defined. In the course of the experiment, FECOs were recorded at 2 frames per second (fps) and continuously monitored. A change of the FECO fringe position (which is, in fact, a wavelength shift Δλ away from the initial λ_0_) can be correlated to a shift in distance, Δ*D*, of the opposing mirrors.

*Electrochemistry*: The electrolyte was 1 m LiPF_6_ salt dissolved in ethylene carbonate equally mixed by volume with diethylene carbonate, with additional fluoro‐ethylene carbonate (10 wt%) and vinylene carbonate (2 wt%). As a counter and reference electrode, a Li foil was used.

*XPS*: The used XPS system at the Department of Physical Chemistry at the University of Oldenburg was an ESCALAB 250 Xi (Thermo Fisher) with an Al monochromatic X‐ray source. An Ar‐filled glovebox was directly attached to the XPS system in order to measure battery electrodes after cycling or opening of Swagelok‐type cells.

## Conflict of Interest

The authors declare no conflict of interest.

## Supporting information

Supplementary
