# Supplementary material for: In Situ Mechanical Analysis of the Nanoscopic Solid Electrolyte Interphase on Anodes of Li‐Ion Batteries
Source: Adv Sci (Weinh). 2019 Jun 14;6(16):1900190. doi: 10.1002/advs.201900190 (PMC6702625; doi:10.1002/advs.201900190)
Supplement: Supplementary file 1 — Supplementary [file ADVS-6-1900190-s001.pdf]

Copyright WILEY-VCH Verlag GmbH & Co. KGaA, 69469 Weinheim, Germany, 2019.

Supporting Information

for *Adv. Sci.,* DOI: 10.1002/advs.201900190

In Situ Mechanical Analysis of the Nanoscopic Solid Electrolyte Interphase on Anodes of Li-Ion Batteries

Boaz Moeremans, Hsiu-Wei Cheng, Claudia Merola, Qingyun Hu, Mehtap Oezaslan, Mohammadhosein Safari, Marlies K.Van Bael, An Hardy, Markus Valtiner,* and Frank Uwe Renner**

Copyright WILEY-VCH Verlag GmbH & Co. KGaA, 69469 Weinheim, Germany, 2019.

Supporting Information

In-Situ Mechanical Analysis of the Nanoscopic Solid Electrolyte Interphase on Anodes of Li Ion Batteries

Boaz Moeremans, Hsiu-Wei Cheng*, Claudia Merola, Qingyun Hu, Mehtap Oezaslan, Mohammadhosein Safari, Marlies K. Van Bael, An Hardy, Markus Valtiner*, Frank Uwe Renner*

In the first discharge, between 2.7 and 2 V vs. Li, we observe a clear cathodic activity at the Au electrode which disappears upon further cycling (Figure 2). Aurbach et al., [36] have observed a similar phenomenon and a cathodic peak around 2.8 V vs. Li. They suggested that this peak is a consequence of reactions following an initial reduction of water trace impurities

$H_2O + Li^+ + e^- = LiOH + ½ H_2$ (Eq. S1)

We conduct a differential capacity analysis in order to highlight different steps in the SEI formation (Figure S1). Upon discharge below 2V, a cathodic peak is observed around 1.54 V (Figure S1(b)) and fades away in the subsequent cycles (Figure S1(c-d)). This peak might be attributed to the reduction of dissolved oxygen traces [1]. A minor cathodic peak can be detected at 1.13 V (Figure S1(b)) which is assigned to the water reduction (Eq. S1). LiOH is insoluble in the electrolyte and hence expected to deposit on the Au. The most significant cathodic peak shows up at 0.46 V (Figure S1(b)) and remains significant for all the cycles Figure S1(e-f). This peak is well located in the potential range suggested in the literature for SEI formation [37-39] and is supported by the onset of a visible mirror shift below 1.5 V (Figure 2). The differential capacity analysis provides an additional illustration of the processes at play.

The SEI film is generally recognized as having a very complex structure of which determination of the exact chemical composition is not trivial. Here, however, we use the mirror-shift data together with the electrochemical results (Figure 2) in order to conduct a simple kinetic analysis of the SEI growth. This analysis in turn sheds more light on the formulation of the SEI layer.

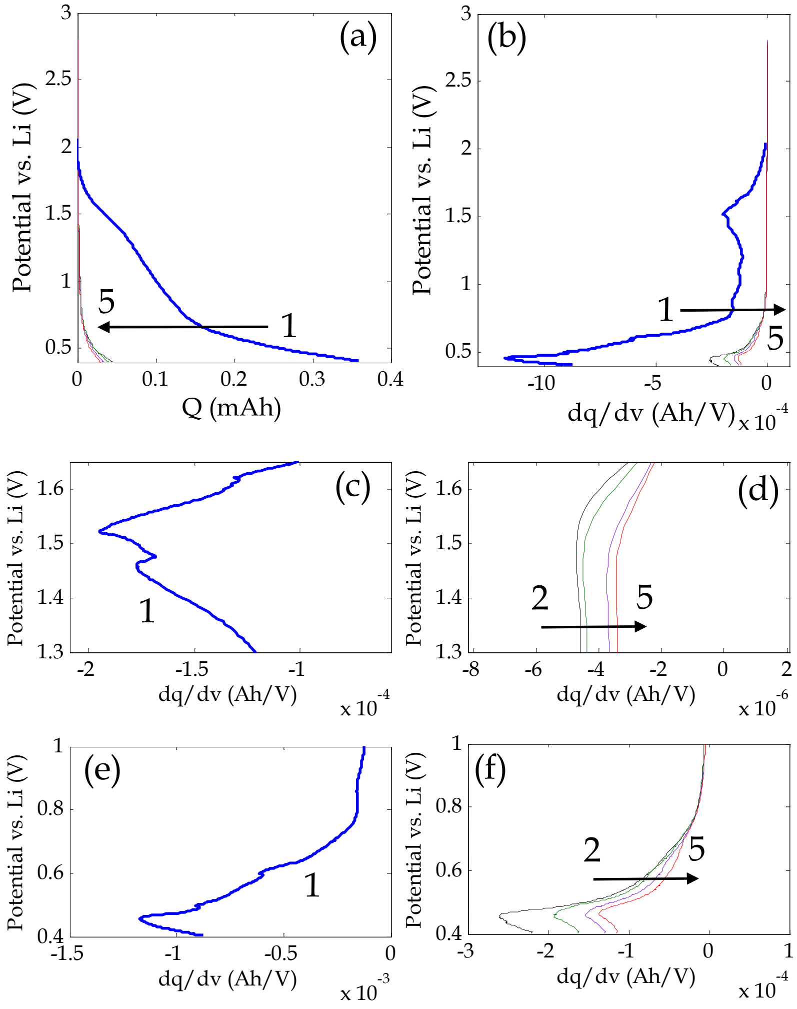

Supporting Display Figure S1 (a) Discharge curves and (b-f) corresponding differential capacity profiles for the five consecutive galvanostatic cycles (Figure 2).

The rate of SEI growth can be simply related to the Faradaic cathodic current according to

$$\frac{d\delta}{dt} = \frac{I}{AnF}\vartheta \qquad \text{(Eq. S2)}$$

where, δ denotes SEI length, I current, A interfacial reaction area, F Faraday constant, n stoichiometry of Li in the SEI product, and ϑ the molar volume of the SEI. We can further rearrange Eq. S2 in order to solve for ϑ in a discrete manner for every discharge cycle (ϑ_i)

$$\vartheta_i \approx \frac{An_iF}{\Delta Q_i}\Delta\delta_i\,,\ (i=1:5) \qquad \text{(Eq. S3)}$$

where, i denotes the discharge cycle and ΔQ represents the accumulated Faradaic charge involved in the SEI formation. This quantity is estimated after subtraction of capacity contributions experienced during charge, that approximates the non-faradaic and non-SEI contributions to the overall current

$$\Delta Q_i = |\int Idt|_{\text{discharge},i} - |\int Idt|_{\text{charge},i}\ , \quad (i{=}1{:}5) \qquad \text{(Eq. S4)}$$

The estimated ϑ as a function of cycle number is presented in Figure S.2. For every cycle, two sets of ϑ are reported based on the values assumed for n (green and purple bars for n=1 and n=2, respectively). In the same figure, the molar volume of most-relevant known SEI candidates (ϑ^*) are superimposed as horizontal lines for comparison [39]. We determine the composition of SEI increment ($\Delta\delta$) over each cycle following a comparison between ϑ_i and ϑ^*(Figure S.3).

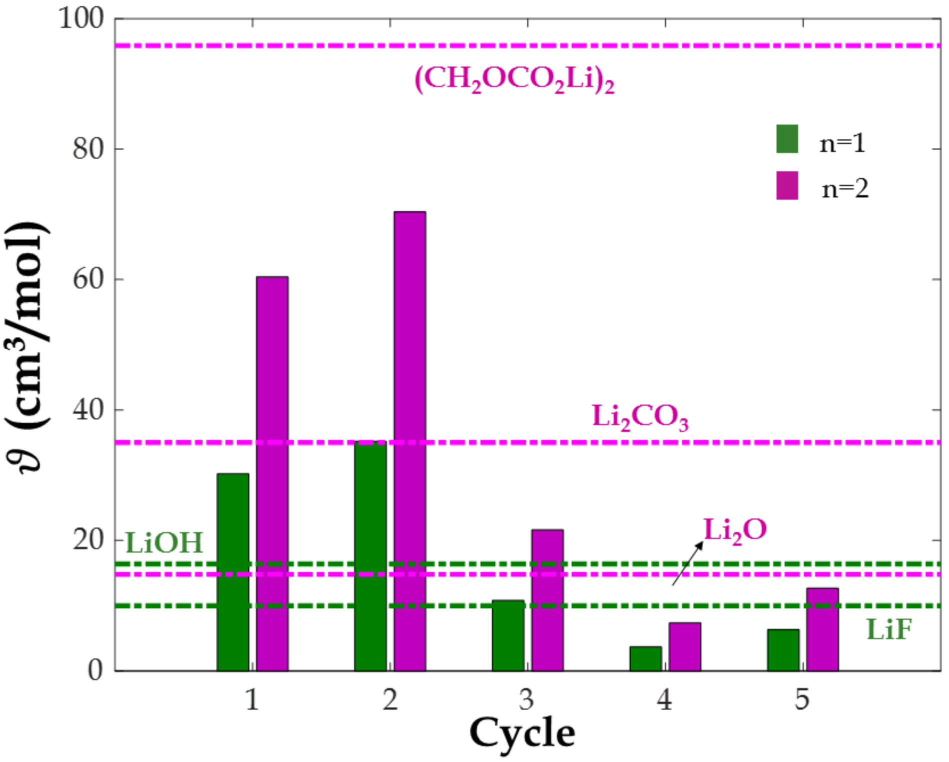

Supporting Display Figure S2. Estimated molar volume of the SEI increments (purple and greens bars) over the 5 cycles together with those of known SEI components (horizontal lines).

It is a common practice to summarize the dynamics of SEI growth in a so called power law [41-43],

$$\delta(t) - \delta(0) = bt^{\alpha} \qquad \text{(Eq. S5)}$$

where, t is time, b a constant, and α is a time dependent exponent. Figure S.4 presents the variation of α over the course of 5 cycles. The observed trend is in good agreement with the theoretical predictions [41-43] and suggest that SEI growth is mostly limited by the transport of electrons and/or solvent species. Here, α levels off around 0.3 which is below the expected value of 0.5 for an ideal diffusion-limited mode of growth [41, 43].

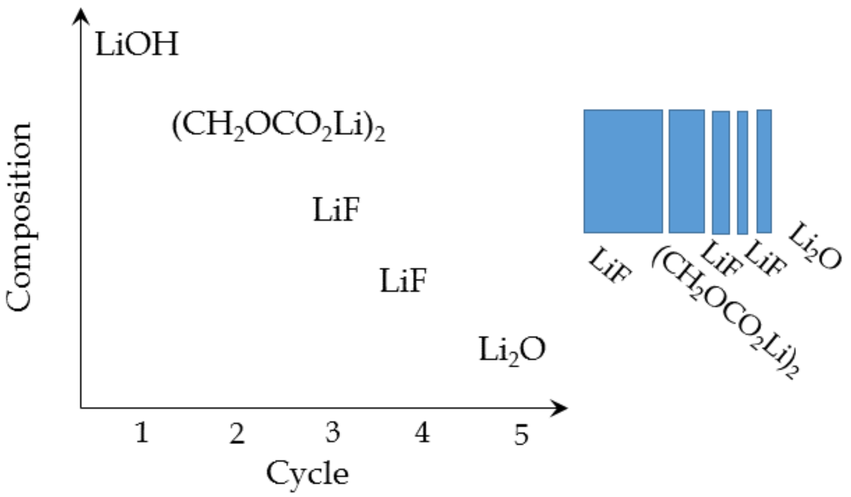

Supporting Display Figure S3. Proposed composition of SEI increments over five cycles based on the results presented in Figure S2.

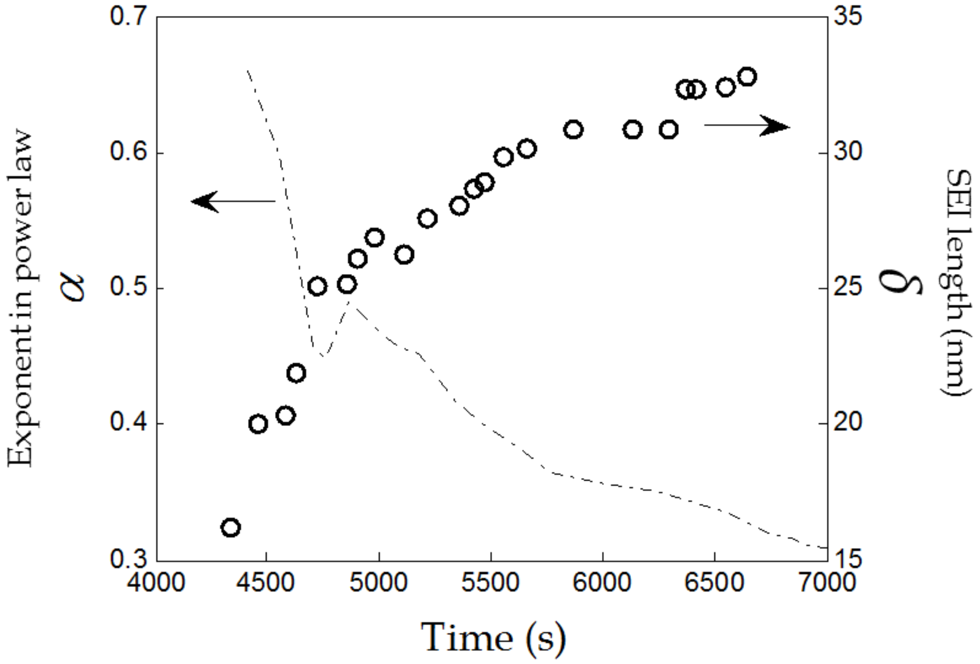

Supporting Display Figure S4. Evolution of the exponent in the typical power law for the SEI growth over 5 cycles (following data from Figure 2)

References

36. D. Aurbach, M. Daroux, P. Faguy, E. Yeager, 'The electrochemistry of nobel metal electrodes in aprotic organic solvents containing lithium salts,' J. Electroanal. Chem., 297 (1991) 225-244.

37. S. Mori, H. Asahina, H. Suzuki, A. Yonei, and K. Yokoto, 'Chemical properties of various organic electrolytes for lithium rechargeable batteries: 1. Characterization of passivating layer formed on graphite in alkyl carbonate solutions,' J. Power Sources, 68 (59) (1997).

38. D. Aurbach, E. Zinigrad, Y. Cohen, and H. Teller, 'A short review of failure mechanisms of lithium metal and lithiated graphite anodes in liquid electrolyte solutions,' Solid State Ionics, 148, 405 (2002).

39. A. Naji, J. Ghanbaja, B. Humbert, P. Willmam, and D. Billaud, 'electroreduction of graphite in LiCLO4-ethylene carbonate electrolyte. Characterization of the passivating layer by transmission electron microscopy and Fourier-transform infrared spectroscopy,' J. Power Sources, 63, 33 (1996).

40. D. Aurbach, Y. Ein-Eli, O. Chusid, Y. Carmeli, M. Babai, H. Yamin, 'The correlation between the surface chemistry and the performance of Li-carbon intercalation anodes for rechargeable 'rocking chair' type batteries,' J. Electrochem. Soc., 141 (3) (1994) 603-611.

41. E. Peled, 'The electrochemical behavior of Alkali and Alkaline earth metals in nonaqueous battery systems – the solid electrolyte interphase model,' J. Electrochem. Soc., 126 (1979).

42. J. Christensen and J. Newman, 'A mathematical model for the lithium-ion negative electrode solid electrolyte interphase,' J. Electrochem. Soc., 151 (2004).

43. M. Safari, M. Morcrette, A. Teyssot, C. Delacourt, 'Multimodal physics-based aging model for life prediction of Li-ion batteries,' 156 (3) (2009).
